# Editorial: SOCS: regulation of the immune system at a whole new level

**DOI:** 10.3389/fimmu.2025.1588549

**Published:** 2025-03-26

**Authors:** Sandra E. Nicholson, Chulbul M. Ahmed, Karen Doggett, Daniela Marasco, Howard M. Johnson

**Affiliations:** ^1^ Walter and Eliza Hall Institute of Medical Research, The University of Melbourne, Parkville, VIC, Australia; ^2^ Department of Microbiology and Cell Science, University of Florida, Gainesville, FL, United States; ^3^ Department of Pharmacy, University of Naples Federico II, Naples, Italy

**Keywords:** SOCS1, SOCS2, CISH, SOCS3, immune checkpoint, interferon, E3 ligase, SH2 domain

Immune checkpoints orchestrate a complex regulatory network, tasked with keeping the immune system in check to avoid autoimmune and inflammatory diseases. The field is dominated by the development of new checkpoint blockers to treat cancer and infectious diseases, following the success of antibody therapies targeting programmed cell death 1 (PD-1), its ligand PD-L1 or cytotoxic T-lymphocyte-associated antigen 4 CTLA4, which have revolutionized cancer treatment. Regulatory T cells (Tregs) and members of a protein family of intracellular regulators called Suppressors of cytokine signaling (CIS, SOCS1, SOCS2 and SOCS3) play central roles to limit the immune response. SOCS1 is a key checkpoint in the immune response to cancer and infection, SOCS3 in various inflammatory diseases, and together they allow crosstalk among cytokines in non-immune and immune cells. CIS (CISH gene) is a critical checkpoint in Natural killer (NK) and T cell control of cancer, while SOCS2 has been reported to have roles in controlling allergy and cancer, including acute myeloid leukemia. Many of these regulatory players are interconnected and interdependent. For example, SOCS1 was shown to stabilize Foxp3+ Treg peripheral cells. There is also evidence that SOCS1 can regulate other checkpoints such as PD-L1, via inhibition of interferon gamma (IFNγ) signaling. Thus, manipulation of SOCS activity or levels could influence other checkpoint factors, and/or synergize with current immune checkpoint blockers. Given that the SOCS proteins are intracellular regulators, the challenge is to positively and negatively regulate these molecules in an informative way with potential for therapeutic translation.

The interest in CIS and SOCS1 as powerful immune checkpoint modulators, is evidenced by an active Phase I/II clinical trial using autologous CISH-deleted tumor infiltrating lymphocytes (TILs; NCT04426669) in gastrointestinal cancer, and recruitment for two new trials using SOCS1-deleted TILs (NCT06237881/NCT06237881). Despite this, there appears to be a widespread lack of awareness that SOCS are key immune checkpoints. This was reflected in a recent call for innovative immune checkpoint inhibitors, which neglected to mention the SOCS system. This may be because unlike PD-1 and CTLA-4, which can be targeted by antibodies, regulation of SOCS activity presents a unique challenge due to their intracellular location, and the inherent difficulty of targeting the conserved SH2 or SOCS box function. Contrary to this perception, there are multiple strategies, including gene editing, anti-sense oligonucleotides, microRNAs and intracellular antibodies, that could be used to manipulate SOCS function.

This Research Topic includes 8 reviews and one primary research article that explore various cytokine and SOCS-related disorders, examining different approaches to regulate SOCS protein activity. However, these papers are just the tip of the iceberg. The recent development of a chemical SOCS2-SH2 inhibitor (Lynch et al.) offers hope that the SOCS-SH2 domain is not as intractable as previously thought. Effective SOCS1 and SOCS3 peptidomimetic agonists and antagonists have been developed with potential for treatment of viral and bacterial infections, cancer, autoimmune and inflammatory diseases such as uveitis, as well as the cellular dysregulation associated with type 2 diabetes. In particular, a cell penetrating peptide mimicking the SOCS1/3 kinase inhibitory region (KIR) has potential for treatment of several blinding diseases including uveitis, diabetic retinopathy, glaucoma and macular degeneration (Stafford et al., Ahmed et al., Cugudda et al., Johnson & Ahmed).

Haploinsufficiency for loss-of-function SOCS1 variants is associated with inflammatory autoimmune diseases such as rheumatoid arthritis, SLE and psoriasis, while in mice, SOCS1 restraint of inflammatory signaling is critical for post-natal survival (Bidgood et al.; Korholz et al.). Specifically, SOCS1 knockout mice, although born viable, only survive for a few weeks as SOCS1 is a key regulator of the cytokine IFNγ, which in the absence of SOCS1, drives a fatal systemic inflammatory disease. Reconstitution of SOCS1 knockout mice with a SOCS1 mimetic containing the kinase inhibitory region (KIR) together with CD4^+^ T cells is partially protective; providing some insight into the complex interactions between regulatory factors, including cytokines, immune cells, and SOCS1 that are required for homeostasis. Importantly, SOCS1 and SOCS3 have been shown to be intrinsic virulence factors, capable of enhancing viral diseases by suppression of type I interferon signaling. A peptide corresponding to the activation loop of JAK2 (residues 1001-1013) has been shown to counteract SOCS1/3 induction, and was similarly found to have broad antiviral activity against a variety of viruses in animal models (Ahmed and Johnson).

Interestingly, loss of SOCS1 and SOCS3 results in skin inflammation, and has been associated with an increased rate of cancer onset (Morelli et al.). SOCS1 in particular can both restrict anti-tumor immunity and act as a tumor suppressor, depending on the cellular and disease context (Bidgood et al., Ilangumaran et al.). SOCS1 has roles in CD4^+^ and CD8^+^ T cells, dendritic cells and macrophages that limit anti-tumor immunity. SOCS1 inhibition of IFNγ-driven MHC-I expression may also increase immune evasion, while paradoxically, SOCS1-associated proteasomal activity may affect antigen processing and presentation, resulting in enhanced immune recognition. The loss of SOCS1 by epigenetic or micro-RNA mediated mechanisms has also resulted in tumor progression in several cancers (Korholz et al.).

Although this Research Topic has a strong focus on SOCS1, we should not forget the important role of SOCS3 in limiting inflammatory signaling downstream of the gp130 cytokine family, or the role of CIS in regulating IL-15, GM-CSF and T cell receptor signaling. No doubt there are additional roles for this important family of negative regulators that are yet to be discovered, and opportunities to take advantage of these cellular inhibitors of signaling for the development of new therapies in the treatment of inflammatory disease.

